# A multimodal AI-based non-invasive COVID-19 grading framework powered by deep learning, manta ray, and fuzzy inference system from multimedia vital signs

**DOI:** 10.1016/j.heliyon.2023.e16552

**Published:** 2023-05-25

**Authors:** Saleh Ateeq Almutairi

**Affiliations:** Taibah University, Applied College, Computer Science and Information department, Medinah, 41461, Saudi Arabia

**Keywords:** Audio classification, COVID-19, Chest X-ray, Deep learning (DL), Fuzzy logic, Manta-ray foraging algorithm (MRFO), Metaheuristic optimization, SMOTE oversampling

## Abstract

The COVID-19 pandemic has presented unprecedented challenges to healthcare systems worldwide. One of the key challenges in controlling and managing the pandemic is accurate and rapid diagnosis of COVID-19 cases. Traditional diagnostic methods such as RT-PCR tests are time-consuming and require specialized equipment and trained personnel. Computer-aided diagnosis systems and artificial intelligence (AI) have emerged as promising tools for developing cost-effective and accurate diagnostic approaches. Most studies in this area have focused on diagnosing COVID-19 based on a single modality, such as chest X-rays or cough sounds. However, relying on a single modality may not accurately detect the virus, especially in its early stages. In this research, we propose a non-invasive diagnostic framework consisting of four cascaded layers that work together to accurately detect COVID-19 in patients. The first layer of the framework performs basic diagnostics such as patient temperature, blood oxygen level, and breathing profile, providing initial insights into the patient's condition. The second layer analyzes the coughing profile, while the third layer evaluates chest imaging data such as X-ray and CT scans. Finally, the fourth layer utilizes a fuzzy logic inference system based on the previous three layers to generate a reliable and accurate diagnosis. To evaluate the effectiveness of the proposed framework, we used two datasets: the Cough Dataset and the COVID-19 Radiography Database. The experimental results demonstrate that the proposed framework is effective and trustworthy in terms of accuracy, precision, sensitivity, specificity, F1-score, and balanced accuracy. The audio-based classification achieved an accuracy of 96.55%, while the CXR-based classification achieved an accuracy of 98.55%. The proposed framework has the potential to significantly improve the accuracy and speed of COVID-19 diagnosis, allowing for more effective control and management of the pandemic. Furthermore, the framework's non-invasive nature makes it a more attractive option for patients, reducing the risk of infection and discomfort associated with traditional diagnostic methods.

## 1 Introduction

**Figure 1:**
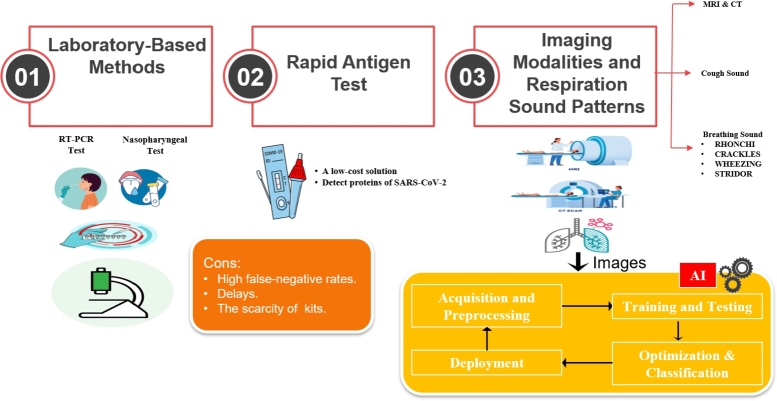
Testing approaches to diagnose and manage COVID-19 patients.

Recently, people worldwide have been vulnerable to numerous ongoing pandemics, such as monkeypox and coronavirus disease (COVID-19). Unfortunately, COVID-19 remains a deadly pandemic claiming over 621 million confirmed cases and 6.5 million deaths worldwide [1]. In addition, COVID-19 dramatically impacts the healthcare system, social activities, safety, and the economy. As a result, it is critical to discover COVID-19 as quickly and accurately as possible to slow the disease's spread. Fig. 1 shows three testing approaches to diagnose and manage COVID-19 patients: laboratory-based methods, Rapid Antigen Test (RAnT), and image modalities-based.

Laboratory-based methods such as nasopharyngeal swab test and reverse transcription-polymerase chain reaction RT-PCR are the common gold standard that detects the specific genetic material of the virus. However, these laboratory-based tests are time-consuming and require expert laboratory technicians and clinical facilities [2]. On the other hand, the RAnT is a low-cost solution that uses nasopharyngeal swabs to detect proteins of SARS-CoV-2. However, the main challenges for these methods are the high false-negative rates, on-site testing, and the scarcity of Kits, especially in underdeveloped countries. As a result, COVID-19-infected patients are frequently screened using chest imaging modalities such as chest X-rays (CXR) and computed tomography (CT) scans. Also, cough and respiration sound patterns are employed as a first-line investigation to detect COVID-19. As a result, the analysis of suspected patients' chest images offers a tremendous promise for screening, and early COVID-19 diagnosis [3]. Unfortunately, the diagnosis depends on the radiologists' experience, which causes some issues. It requires a long time to diagnose chest imaging because it contains hundreds of slices. Furthermore, because COVID-19 shares characteristics with other types of pneumonia, radiologists must have sufficient expertise to differentiate similar diseases.

Recently, there has been a significant increase in scientific community cooperation [4] for developing cost-effective, rapid, and accurate diagnostic approaches that widely benefit from computer-aided diagnosis (CAD) systems and artificial intelligence (AI). In this vein, the recent advances in CAD systems and AI have led to the emergence of machine learning and deep learning (DL) as common tools for automatic medical image prognosis and classification of different diseases. For example, concerning COVID-19, medical images and cough and breathing sound patterns can be analyzed to identify abnormalities.

Recently, medical image diagnosis based on CXR image analytics has been gaining popularity for COVID-19 detection. CXR is widely available and economical, besides the automatic diagnosis based on CXR images has advantages over radiologists' examination, including repeatability and improvements too subtle to be noticed visually. Also, cough sounds can be used as pre-screening tests for infected patients. Coughing sounds can be converted to image vectors using DL approaches then these images can be analyzed by a CAD system to detect respiratory abnormalities like fine crackles and coarse crackles remotely. Cough sound classification utilizing artificial intelligence is an urgently needed area of research [5]. Thus, DL models can be applied to medical image analysis to accurately diagnose COVID-19 and mitigate the shortage of skilled medical staff. Accordingly, it represents an effective way for telemedicine that can control and prevent this pandemic scenario.

Deep learning (DL) technology has assisted radiologists in detecting various respiratory problems with high diagnostic accuracy through medical imaging [6], [7]. Medical datasets, particularly those with many training samples, lend themselves well to deep learning. Transfer learning (TL) is a DL technique that is trained on one problem and applied to another. Transfer learning (TL)can be used for two objectives: 1) switching either supervised or unsupervised features from the original problem and 2) retraining only unlocked layers while avoiding overtraining for the target task [8]. Deep neural network efficiency is highly dependent on the values of their hyperparameters [9]. Also, the automatic diagnosis of cough disease is a hot research topic. The CAD systems for COVID-19 detection, via cough and breath sounds, support the health service. Accordingly, the virus spread is limited by monitoring infected patients remotely regularly.

According to the literature review, most DL model research uses pre-trained deep-learning architectures to diagnose COVID-19 based on cough sounds and medical images. Furthermore, most studies generally focus on diagnosing COVID-19 based on a single modality. The research gap in this review are as follows: (1) Due to the virus's constant gene mutation, CXR images alone cannot accurately detect the virus in its early stages [10]. (2) On the other hand, models for detecting COVID-19 based on cough sounds demonstrate a low level of accuracy. (3) Most proposed DL methods manually determine the optimal configurations for the models. This study suggests a multimodal classification framework for COVID-19 that achieves trusted classification.

The main objective of this study is to introduce a four-layer diagnostic framework for COVID-19 based on elementary patient diagnosis, cough profile, and CXR. Furthermore, a fuzzy logic inference system is used to report an accurate decision. The contributions of this study can be summarized in the following points:

- Propose a framework for automatic and accurate COVID-19 classification based on multimedia Vital Signs using CNN, TL, FIS, and MRFO Algorithms.
- Four cascade non-invasive diagnostic phases to reliably determine if a patient is infected with COVID-19.
- The hyperparameters are optimized using the state-of-the-art Manta-Ray Foraging Algorithm (MRFO), which allows for reporting optimal performance data.
- To correct the problem of underrepresentation, the synthetic Minority Oversampling Method (SMOTE) is used to perform oversampling.
- The suggested method is flexible since the hyperparameter values of the CNN architecture are automatically determined.
- Concerning accuracy, DenseNet201 provides the highest performance metrics.
- Four models suggest a ratio of more than 50% for TF learning, and four models offer a percentage greater than 40% for dropout.
- Tests are conducted five times to obtain validated results, and the CI for each statistic is measured.

The rest of the paper is organized as follows: Section 2 introduces the background of computer-aided decision support systems. Next, section 3 reviews the related studies of COVID-19 classification based on cough profiles and CXR images. Then, Section 4 describes the COVID-19 diagnostic framework consisting of four layers, Section 6 highlights the study limitations, and Section 5 discusses the experimental results. Finally, the paper is concluded in Section 7.

## 2 Background

Rapid growth has been observed in using deep learning (DL) architectures in medical imaging, including computer-assisted diagnosis systems and medical image analysis, enabling more accurate screening, diagnosis, prognosis, and treatment of numerous relevant disorders [11], [12], [13]. In Deep Learning, a model can be executed from start to finish utilizing only the input data without the requirement to extract features. Recently, machine learning has emerged as a prominent diagnostic tool for clinicians, providing them with an auxiliary instrument [14]. DL techniques have introduced sophisticated image classification without the need for human-engineered features. As a result, DL techniques have gained widespread acceptance for various classification tasks relating to image processing and healthcare applications [15]. Convolutional neural networks (CNNs) have greatly assisted in resolving classification and segmentation problems in CT scans, among other applications [16]. CNN is considered one of the greatest deep learning models due to its use in computer vision, speech recognition, and medical diagnosis. AlexNet, a deep CNN trained with just supervised data, performed well in the ImageNet LSVRC-2012 competition. Alex et al. used dropouts, pooling, and local response normalization to improve CNN training and performance [15], [17].

Current research on the classification of medical datasets reveals substantial constraints, notwithstanding the effectiveness of the applications mentioned above. There is much writing on data imbalances between classes since insufficient training data exists. High accuracy under these settings is no guarantee of excellent illness identification [15], and the variability of the data makes it unlikely that DL models would be trained well. There is a shortage of available COVID datasets, for instance. If the CNN memorizes the discriminant features of the COVID images instead of learning them, overfitting must be avoided or minimized during training. Similar computational resources are needed for CNN inference [16].

Up till now, DL techniques have been implemented in isolated environments. These algorithms are “trained” to handle specific problems. When the distribution of the feature space changes, the models must be rebuilt. Transfer learning is using a model developed for one purpose as the basis for developing a model for another. More refined adjustments to the new model are possible by adding more training data and modernized neural layers [18]. The availability of models trained on diverse datasets and the possibility of transferring layers between DL models for different tasks indicates a promising area of research. On the one hand, we gain from the pre-trained model weights, which make the learning process considerably more efficient; in other words, the model converges quickly because its weights are initially stable. On the contrary, it drastically reduces the amount of labeled data necessary for model training.

By learning from abstract representations, CNNs may evaluate images with a high level of semantics. CNN utilizes filter banks instead of manually generated ones to optimize the image texture. It is well acknowledged that the availability of enormous amounts of data is one of the literature's limitations. The practical use of the technologies mentioned above is unreliable since DL models perform better with more datasets. In addition, these models are constructed using standard parameters. The dataset and the hyperparameters chosen determine the classification performance of a CNN. The hyperparameter parameters have a significant effect on a CNN's training phase. It is essential to remember that model weights and hyperparameters differ. The former is calculated beforehand, whereas the latter is enhanced by training [19]. Consequently, other approaches can be employed to set hyperparameters. The manual selection method is the default because there are so many different configurations, but it should be avoided wherever possible. Similarly, approaches such as grid search do not utilize past evaluations; thus, much time is wasted evaluating inefficient hyperparameter selections. In contrast, Bayesian strategies, which use past evaluation results to map hyperparameters to the likelihood of a score on an objective function, look more effective [16]. Application-dependent hyperparameter selection can result in inferior performance metrics. Rather than randomly selecting hyperparameter values, application-specific values are determined using an optimization technique [20].

Practical engineering problems generally have numerous locally optimal solutions; therefore, deterministic algorithms struggle with them. However, high-dimensional search spaces demand precise optimization. With search spaces expanding exponentially with problem size, thorough searches are unfeasible. In contrast to deterministic algorithms, metaheuristic algorithms can generate an approximate answer without the exact solution. Consequently, computations can be greatly streamlined. In addition, randomness introduced by meta-heuristic algorithms can eliminate the local optimal problem. These advantages apply to metaheuristic approaches to global optimization problems. In the past decade, numerous Swarm intelligence algorithms, such as the Particle Swarm Optimization Algorithm, the Krill Herd Optimization Algorithm, and the Beetle Antenna Search Algorithm, have been proposed [21]. Search heuristics are required to effectively explore the solution space, which can be vast depending on the number of layers, size, shape, type, number of neurons, intermediate processing units, and other structural properties.

### 2.1 Metaheuristics

Metaheuristics explore large sets of solutions with less processing power than calculus-based or simple heuristics. Adaptability, self-learning, flexibility, unpredictability, mutually balanced intensifications, diversifications, intuition, robustness, and efficiency evolved to produce intelligent behavior and biological phenomena. Bio-inspired computation is one of the AI fields with the most study. The most recent accomplishment in this field was the development of Swarm Intelligence (SI). Swarm Intelligence, a subfield of bio-inspired computation, is founded on the collective intelligence of enormous populations of animals with basic communication and interaction patterns. Ant colony optimization (ACO), particle swarm optimization (PSO), cuckoo search (CSA), elephant herding optimization (EHO), whale optimization algorithm (WOA), and Manta-Ray Foraging Algorithm (MRFO) are common SI methods (SSO). A highly useful taxonomy of natural optimization approaches contains 132 separate entries [22]. The study [23] overviews evolutionary algorithms and their engineering applications. In [24], the authors present a variety of statistical methods for evaluating meta-heuristic and heuristic optimization strategies. These articles utilize statistical methods to establish credible comparisons between various algorithms.

The Manta Ray Foraging Optimization (MRFO) algorithm models the food-seeking behaviors of manta rays, such as chain foraging, cyclone foraging, and somersault foraging [25]. The manta ray is an aquatic animal. Manta rays lack sharp teeth; hence plankton is their primary source of nutrition [26]. As a result, they devour numerous planktons (about 5 kilograms) per day [27]. The body of a manta ray is flat from top to bottom, and it uses its two pairs of pectoral fins to swim and fly like a bird. In addition, they have forward-extending vertical lobes in front of their enormous terminal mouths. Manta rays are fascinating because of their foraging habit; they may roam alone or in groups of up to fifty, but feeding in groups is the norm [28].

Manta ray foraging optimization (MRFO) is one of the recently published bioinspired metaheuristic algorithms with the potential to solve multiple engineering optimization challenges. MRFO is a suitable method for defining unknown model parameters of commercial photovoltaic units, solving economic power and heating dispatch problems, tracking maximum power points in solar cells, and maintaining thermo-economic optimization of air-fin coolers [29].

Manta rays employ a chain technique to approach food sources by aligning themselves head-to-tail. MRFO's area with the greatest concentration is regarded as the best. Chains of foraging manta rays mimic the natural process of capturing food [26]. They seek to grab prey that was missed or undetected by the preceding Manta. MRFO considers that the best solution to date is an increase in the concentration of plankton, the prey of importance for manta ray chains. The algorithm adjusts the current position of each manta ray depending on the optimal prey target and the manta ray that stands in front of the current manta ray. In this manner, the competitive manta rays reduce the risk of losing prey vision while increasing their odds of capturing food.

Additionally, the MRFO adopts the cyclone strategy [29]. The cyclone feeds when the concentration of prey (plankton) is high. By combining its head and tail, the manta ray generates a spiral in the eye of a cyclone, causing the filtered water to rise. In deep water, swarms of plankton draw the attention of manta rays, which create a foraging chain and spiral toward them as they approach. During the cyclone phases of foraging, manta rays follow the manta ray in front of them to maintain continuity, but they also pursue a spiraling path to reach their meal.

The performance of two distinct mechanisms of metaheuristic algorithms, exploration, and exploitation, depends on cycle foraging [25], [30]. In this phase of foraging, using the best plankton as a reference allows for the intensification of fertile regions surrounding the best solution currently in use, increasing the algorithm's exploitation capabilities. Cyclones contribute considerably to the exploration phase by driving the population to relocate to a nearly random place in search space, causing the population to move away from its current position and the optimum prey position. This approach adds to a more diverse global search area by directing users down avenues not previously examined by the algorithm.

Finally, somersaulting foraging is one of the most stunning natural behaviors living organisms perform. Manta rays encircle their prey plankton and spin backward as they ingest them [25], [29]. The MRFO algorithm interprets food as a hub when each agent flips along somersault foraging. MRFO is a search method based on these three strategies for foraging [31]. Thus, agents can improve their exploitation capabilities by adjusting their position to the optimal solution.

In this part, the basic processes of MRFO are described. MRFO models the three feeding techniques of manta rays: chain foraging, cyclonic foraging, and somersaulting foraging. Similar to other swarm-based metaheuristic algorithms, MRFO first produces the initial populations at random. It is then updated using the three methods mentioned above.

### 2.2 Fuzzy inference system

When uncertainty and imprecision exist, precise logic cannot be applied. In fuzzy logic, it is feasible to calculate results by describing partial membership in sets. The purpose of fuzzy logic is to build a computing paradigm based on how humans think since most classes of real-world objects lack clearly defined membership criteria. Several problems have been modeled with fuzzy set theory and inference systems, particularly those involving decision-making with two or more assessment criteria. It has been proposed that fuzzy inference systems can be used to model qualitative and non-numerical assertions. FIS uses fuzzy numbers and operations to express knowledge via the behavior of linguistic variables governed by “IF-THEN” rules [31]. Thus, a FIS consists of:

- A database of IF-THEN rules;
- The membership functions of all fuzzy sets featured in the rules;
- The decision-making model executes inference operations on the rules.
- The fuzzification model converts the results of the inference into a crisp output.

In a fuzzy inference system, the input and output variables might take on various linguistic values (low, medium, and high), which must be expressed as fuzzy numbers. The rule base determines the result, which depends on the input variables' values and the established rules. For example, IF “Fever” is “high” AND “Saturation” is “low,” THEN “Severity” is “high,” where Fever and Saturation are input variables, and Severity is the output variable. The output fuzzy number of each rule is determined by the input variables' values and the implication relation.

## 3 Related studies

This section reviews the most recent literature on COVID-19 classification based on cough sounds and imaging modalities. Loey and Mirjalili [4] developed a two-stage COVID-19 diagnosing model based on the cough sounds of patients. The first phase transforms sound waves into images using the scalogram technique. The second phase involves feature extraction and classification, which are performed using different deep transfer learning models. They used a dataset that contains 1,457 wave cough sounds. Experimental results prove the effectiveness of the ResNet18 model with a classification accuracy of 94.9% using an SGDM optimizer. Verde et al. [32] developed non-invasive framework for COVID-19 detection based on voice signals. Several ML techniques were trained and tested using the Coswara dataset from 166 subjects. The experimental study proved that Random Forest is the best technique for achieving accurate classification with an accuracy of 82%.

Unais et al. [2] developed a multimodal classification framework called Ai-CovScan that performed CXR images and breathing sound analysis. First, the CXR is scanned, and sounds are recorded via a Mobile application. Transfer Learning using InceptionV3 combined with Multi-Layered Perceptron (MLP) was used. Next, they collected breathing abnormality sounds from different sources. The audio files are converted into a spectrogram video, segmented into spectrogram images, and converted to a 2D-image vector using DCNN. Finally, the MLP is used to identify abnormalities due to COVID-19. A large Chest X-ray dataset was used to train and test the Ai-CovScan framework. Experimental results demonstrated that their proposed model records an accuracy of 80% and 99.66% for breathing sound data analysis and the CXR image dataset, respectively.

Ahmadian et al. [9] introduced a binary image classification approach based on DCNN to classify CXR images. Besides, they deployed a dual-stage approach via the boosted salp swarm algorithm (BSSA) for hyperparameters optimization and support vector machine approach to enhance accuracy. A Mendeley repository gathered a dataset containing 912 positive and 912 normal cases. The experimental results reported an accuracy of 98.85%. Ardakani et al. [33] proposed a COVID-19 classification system that consists of four phases: (1) Image acquisition phase, in which HRCT images were collected using the high-resolution protocol. (2) CT scan pre-processing phase, in which CT scans are converted into greyscale, and experienced radiologists review these images. (3) Deep learning phase in which ten well-known pre-trained CNN were used. (4) Optimization using Transfer-learning. Experimental results reported the best accuracy of 99.51% recorded by ResNet-101.

Khan et al. [34] developed a DBHL framework that combines two deep learning frameworks for COVID-19 classification using CXR images. The first is Deep Hybrid Learning (DHL), based on two developed COVID-RENet models. The second is Deep Boosted Hybrid Learning (DBHL), which fine-tunes the COVID-RENet-1 and 2 using transfer learning and data augmentation. The two models' features are combined to form a single enriched feature space and to achieve better COVID-19 classification using an SVM classifier. A novel chest X-ray dataset that contains 6,448 CXR images for COVID-19 patients and healthy people were constructed. Experiments proved that the proposed DBHL framework achieved an accuracy of 98.53%. A study in [35] introduced a lightweight CNN-based model called C-COVIDNet for multi-class COVID-19 classification using CXR images. The CXR image is first preprocessed for feature extraction. An accuracy of 97.5% was achieved based on the experiments performed on three datasets containing 11,343 CXR images.

In the study of Loay Aly and Alotaibi [36], a dual-stage deep learning approach for COVID-19 classification through cough and breath tones was proposed. First, the cough and breath sounds are transformed into images via a Mel-scale spectrogram approach. Then processing, feature extraction, and classification were performed using nine deep transfer models. They used an open-source dataset containing information data from almost 1,600 cases. The ResNet18 model achieved the best accuracy of 99.2%. Next, Kuluozturk et al. [5] introduced an automatic DKPNet41 model for multi-class Cough disease classification. The DKPNet41 model is divided into four phases: feature generation, signal decomposition, feature selection, and classification. They gathered a cough sound dataset that included four diagnostic categories. The DKPNet41 model is characterized by low computational complexity and achieved a classification accuracy of 99.39% using the k-nearest neighbor (kNN) classifier.

Swarup and Anupam [10] developed a modified residual network-based enhancement (ENResNet) scheme for COVID-19 detection using CXR images. First, the CXR images are transformed into residual images using ResNet and normalization. Then, high-level features are extracted, a features map is constructed, and a final enhanced image is obtained. This image accurately depicts the affected area of a COVID-19 patient. Then, a simple CNN-based classification model is used. The results report a classification accuracy of 99.7% and 98.4% for binary and multi-class classification. A study in [37] developed DL framework for automatic binary classification for COVID-19. An image preprocessing algorithm for feature extraction was proposed. Besides, a deep learning algorithm for feature selection was introduced. The experimental study performed using a dataset of 63,839 CT images reports a classification accuracy of 98.49%.

Munish et al. [38] introduced a deep learning binary classification framework to predict COVID-19 infection using CT and CXR images. They testes twenty models using four datasets that contain 2,450 CT and CXR images. The TL CNN model and LSTM-CNN record the best performance. Sixteen experiments were conducted to validate the framework's feasibility. The average accuracy of the ensemble CNN of 96.51% was recorded. Ranjan et al. [39] introduced a lightweight DL LW-CBRGPNet model for COVID-19 multi-class and binary classification using CXR images. The proposed model used CNN architecture and training from scratch without feature extraction. A three classes dataset of 2,250 CXR images was used for conducting experiments. Using various hyperparameters, the proposed model achieved an accuracy of 98.33%.

Table 1 summarizes recent studies on COVID-19 classification using different techniques, including cough sounds, voice signals, and imaging modalities. The studies employed various deep learning models, including ResNet18, InceptionV3, and CNN-based models, and datasets of different sizes, ranging from 166 to 6,448 CXR images. The studies achieved classification accuracies ranging from 80% to 99.66%.

**Table 1:** Summarization of the related studies.

Study	Technique	Model	Dataset	Accuracy
Loey and Mirjalili [4]	Cough sounds	Transfer Learning	1,457 cough sounds	94.9%
Verde et al. [32]	Voice signals	Random Forest	Coswara dataset	82%
Unais et al. [2]	CXR images and breathing sounds	InceptionV3 and MLP	Chest X-ray dataset	80% and 99.66%
Ahmadian et al. [9]	CXR images	DCNN and SVM	1,824 CXR images	98.85%
Ardakani et al. [33]	CXR images	Pre-trained CNN	HRCT dataset	99.51%
Khan et al. [34]	CXR images	DBHL	6,448 CXR images	98.53%
Rajawat et al. [35]	CXR images	CNN-based model	Three datasets	97.5%
Loay Aly and Alotaibi [36]	Cough and breath sounds	Transfer Learning	Almost 1,600 cases	99.2%
Kuluozturk et al. [5]	Cough sounds	DKPNet41	Cough sound dataset	99.39%
Swarup and Anupam [10]	CXR images	ResNet and CNN	COVID-19 dataset	98.38%
Saheb et al. [37]	CT images	Deep Learning	63,839 CT images	98.49%
Munish et al. [38]	CT and CXR images	LSTM-CNN	2,450 CT and CXR images	96.51%
Ranjan et al. [39]	CXR images	DL LW-CBRGPNet	2,250 CXR images	98.33%

## 4 Methodology

**Figure 2:**
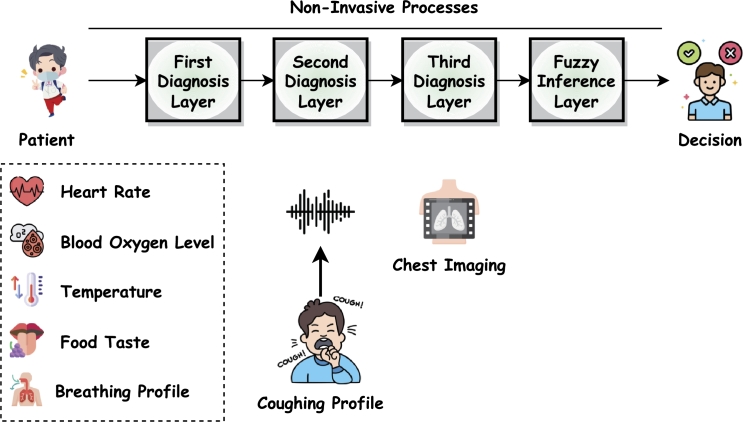
Graphical overview on the suggested 4-layers framework.

The main objective of this research is to develop a non-invasive diagnostic framework for accurately detecting COVID-19 in patients, using four cascaded layers, as depicted in Fig. 2. The suggested methods are designed to work together in a sequential manner, resembling a protocol that clinicians can use to diagnose COVID-19. Each diagnosis layer is intended to filter out non-COVID-19 cases and improve the accuracy of the subsequent diagnosis layers. The first layer performs basic diagnostics such as patient temperature, blood oxygen level, and breathing profile, providing initial insights into the patient's condition. The second layer analyzes the coughing profile, while the third layer evaluates chest imaging data such as X-Ray and CT scans. Finally, the fourth layer utilizes a fuzzy logic inference system based on the previous three layers to generate a reliable and accurate diagnosis. The specific details of each layer and the overall framework are explained in the following subsections.

### 4.1 First diagnosis layer

As mentioned before, the first layer of the suggested non-invasive diagnostic framework initiates the first insight into the patient's case as it handles the elementary diagnosis such as heart rate, temperature, blood oxygen level, food taste, and breathing profile. COVID-19 infected patients have reported a wide range of symptoms ranging from mild to severe, typically appearing 2 to 14 days after virus exposure [40]. These symptoms include fever, dry cough, shortness of breath (i.e., difficulty of breathing), loss of taste and appetite, low blood oxygen levels, arrhythmia, and irregular and fast heart rate. The Chinese CCDC published a paper in the Chinese Journal of Epidemiology on February 17, 2020 [41], concluding that 80.9% of COVID-19 cases are mild with flu-like symptoms while 13.8% infections are severe, resulting in severe symptoms including pneumonia and shortness of breath. Additionally, 4.7% of cases were critical which include septic shock, respiratory failure, and multi-organ failure. Only in about 2% of the reported cases, the virus was fatal.

In Wang et al. [42], 138 hospitalized patients were examined and findings were reported. They found that about 98.6% of cases had experienced fever and 59.4% cases had a dry cough. According to Huang et al. [43], 98% of cases suffered from fever and 76% experienced dry cough where 41 COVID-19 cases were studied. From the Chen et al. study based on 99 patients [44], 83% of cases had a fever, 82% had a cough, and 31% had shortness of breath. Additionally, 90% of cases experienced more than one symptom or sign and 15% experienced cough, fever, along with shortness of breath. Maloberti et al. [45] reported that the most popular arrhythmia associated with COVID-19 infection is sinus tachycardia. In their study, 697 cases of COVID-19 were examined. The mean heart rate at the beginning of the infection was 90 ± 18 bpm and at the discharge state, the mean heart rate decreased by 10 bpm. Only 5.5% of cases had (HR > 100) bpm at discharge. The oxygenation parameters such as SaO2 and SpO2 can be used to assess levels of oxygen in the blood. SpO2 is the arterial oxygen pressure that is detected by a pulse oximeter while SaO2 is the oxygen saturation of arterial blood. The amount of oxygen bound to Hb will increase as the partial pressure of oxygen increases. Normally, SaO2 and SpO2 is > 95% [46]. In Tobin et al. [47], a study on three COVID-19 patients ranging in age from 58 to 74 years was conducted. The SaO2 and SPO2 ranged between 69% and 75% and 68% and 76%, respectively.

From that (1) when tested with a pulse oximeter, people with healthy lungs should have an oxygen level of 80-100 mmHg, or 95% to 100% [48]. (2) Normal body temperature varies according to the individual, age, activity level, and daytime. It is estimated that the average normal body temperature is 98.6 ^∘^F (37 ^∘^C). According to various studies, normal body temperature can range between 97 ^∘^F (36.1 ^∘^C) to 99 ^∘^F (37.2 ^∘^C). A fever of further than 100.4 ^∘^F (38 ^∘^C) is typically caused by an infection (or disease). The temperature of the body changes during the day [49]. (3) Adults typically have a heart rate during rest of 60 to 100 beats per minute [50]. Hence, this study summarizes these insights in Table 2. A patient can be initially considered a COVID-19 patient if the temperature is high, the food taste is low (or medium), the blood O2 level is low (or medium), and the heart rate is high. These rules will be used in the fuzzy inference layer to determine the patient COVID-19 grade accurately.

**Table 2:** Summarization of the medical insights in the first diagnosis layer.

Factor	Case	From	To
Human Temperature (^∘^C)	Cold / Low	28	36
	Normal	36	38
	High / Sick	38	42.5
Pulse (Heart Rate) (PPM)	Low	0	60
	Normal	60	100
	High	100	220
Blood O2 Level (%)	Low	0	50
	Medium	50	90
	Normal	90	100
Food Taste	Low	-	-
	Medium	-	-
	High	-	-

### 4.2 Second diagnosis layer

**Figure 3:**
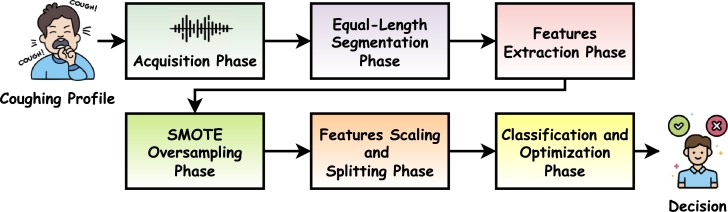
Graphical presentation of the internal phases on the second diagnosis layer.

In the second layer, the patient records a *t*-second coughing audio profile. COVID-19 patients are diagnosed by a dry cough and shortness of breath [51]. There are different state-of-the-art techniques that can be used to analyze the audio waves and classify them such as deep neural networks (DNN) [52]. In short, the current study applies audio features extraction on the coughing profile, preprocesses the features, and classifies them using a deep neural network. Fig. 3 shows the internal phases on the second diagnosis layer.

To perform that, a DNN model is required to be trained and optimized. The current study used the “Cough Dataset” [53] that contains 3,805 train and 5,732 test records. The records are classified into two labels: “COVID-19” (306 cases) and “Normal” (3,499 cases). With regards to the presence of cough in normal individuals within the dataset, it is important to note that coughing can have various causes, such as allergies, environmental factors, or a reflex action to clear the airways, even in individuals without any respiratory conditions or infections.

**Figure 4:**
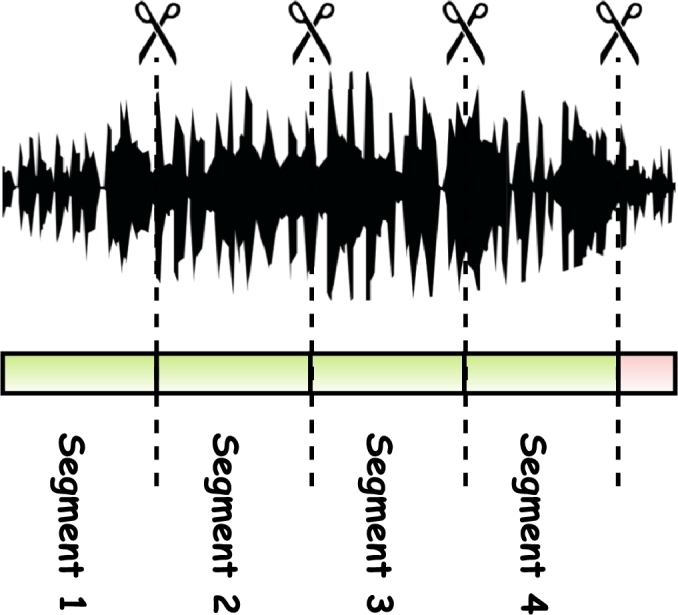
Graphical presentation on the audio segmentation process.

The records do not have a standard (i.e., equal) length (i.e., duration). For example, some records can last for 5 seconds while another some can last for 10 seconds. From these details, **there are two challenges**: (1) the different audio lengths and (2) the dataset imbalanced criteria. Both of these challenges are solved before the training and classification process. Each audio record is segmented into equal 5-second segments and 16 feature types are extracted from each segment as presented graphically in Fig. 4. It shows that an audio wave is segmented into five segments where four are considered and the last is neglected. This happens when the last segmented part length is less than the required length. After applying this step, 9,219 segments are obtained where 8,462 are “Normal” and 757 are “COVID-19” cases. It is worth noting that, denoising is not required as the audio samples were released in high quality and free from any significant noise or distortion.

From the audio records, as noted, 16 feature types are extracted from each segment. They are (1) Mel-Frequency Cepstral Coefficients (MFCC) Slaney [54], (2) MFCC HTK [54], (3) waveform chromagram [55], (4) power spectrogram chromagram [55], (5) constant-Q chromagram, (6) Chroma Energy Normalized (CENS) chromagram [56], (7) mel-scaled spectrogram, (8) spectral contrast [57], (9) spectral centroid [58], (10) spectral flatness [59], (11) p'th-order spectral bandwidth [58], (12) roll-off frequency, (13) Root Mean Square Error (RMSE), (14) zero-crossing rate (ZCR), (15) tonal centroid features (tonnetz) [60], and (16) harmonic tonnetz. In the current study, the FFT window size is set to 2,048 while the number of MFCCs is set to 40. The hop length is set to 512. The dimensions of the calculated features are 9,219×281 where each record has 281 features.

**Figure 5:**
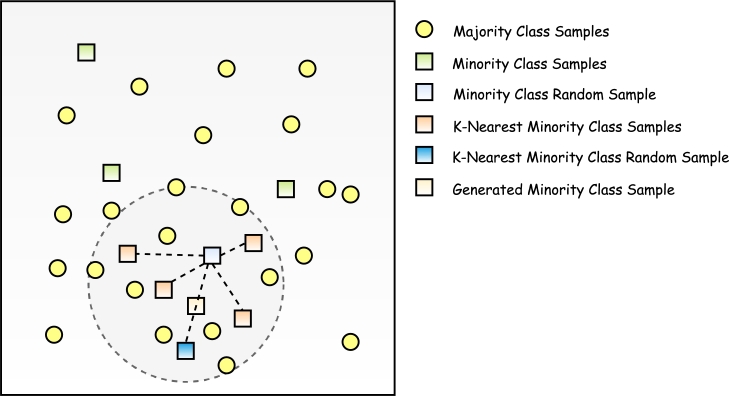
Graphical presentation on the process of generating a minority class sample.

To overcome the imbalanced issue, oversampling is applied using Synthetic Minority Oversampling Technique (SMOTE) [61]. SMOTE is a technique for oversampling which generates synthetic samples for the minority class. This technique assists in tackling the overfitting problem due to random oversampling. It begins by randomly selecting a minority class instance *a* and locating its k-nearest minority class neighbors. The synthetic instance is then created by randomly selecting one of the k-nearest neighbors *b* and connecting *a* and *b* in the feature space to create a line segment. Convexly combining the two selected examples *a* and *b* yields the synthetic instances [62]. Fig. 5 summarizes the process for generating a minority class sample graphically. After applying this step, 16,924 segments are obtained where 8,462 are “Normal” and 8,462 are “COVID-19” cases.

**Figure 6:**
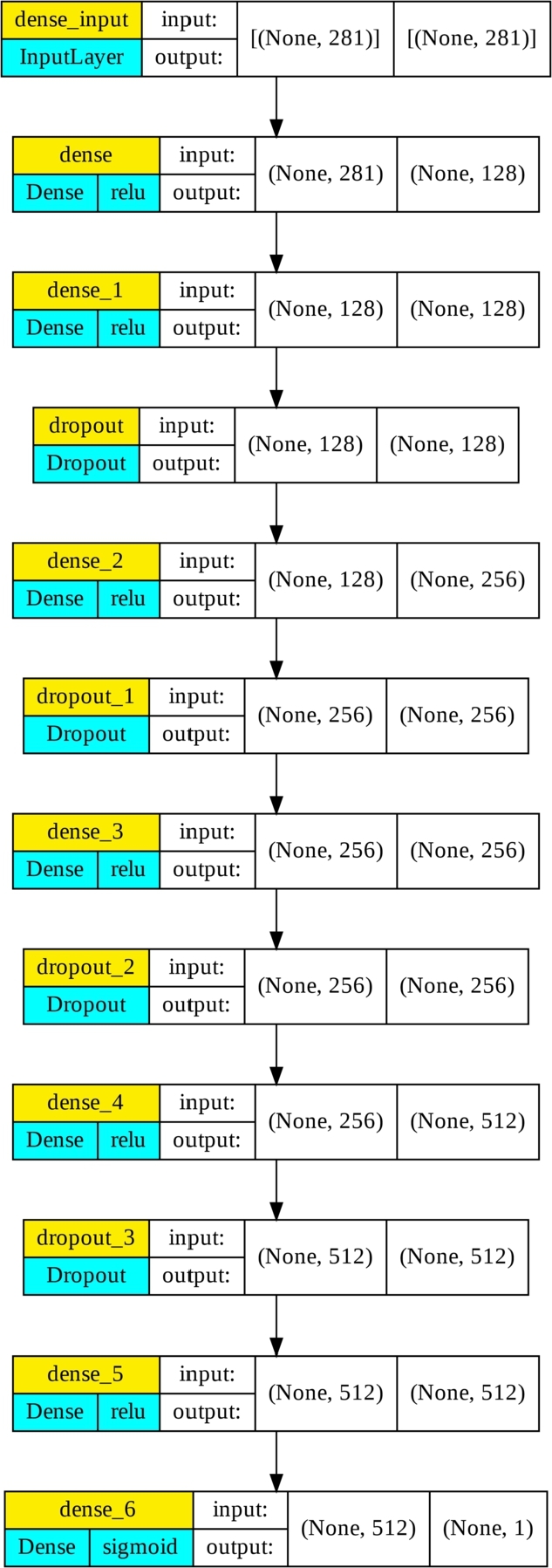
Graphical presentation of the suggested 12-layer DNN architecture in the second diagnosis layer.

The data is now ready to be injected in the classification and optimization phase. As noted, a DNN architecture is used in this phase. It consists of 12 layers as shown in Fig. 6. It consists of (1) an input layer with a size of 281 (the size of the features per record), (2) two dense layers with 128 neurons and ReLU activation function, (3) a dropout layer with a dropout ration of 50%, (4) another dense layer with 256 neurons and ReLU activation function, (5) another dropout layer with a dropout ration of 50%, (6) another dense layer with 256 neurons and ReLU activation function, (7) another dropout layer with a dropout ration of 50%, (8) another dense layer with 512 neurons and ReLU activation function, (9) another dropout layer with a dropout ration of 50%, (10) another dense layer with 512 neurons and ReLU activation function, and (11) an output layer with a Sigmoid activation function. The utilized loss function is binary cross-entropy and the utilized parameters optimizer, Adam, has a learning rate of 0.0005. Adam optimization is a gradient descent stochastic method that uses an adaptive estimation of first and second-order moments [63]. The data is split into 80% for training (and validation) and 20% for testing. The data is also scaled using the min-max scaler (Equation (1)) so the features are ranged ∈[0,1]. The training hyperparameters are (1) 1,500 epochs, (2) three batch sizes: 256, 512, and 1,024, and (3) 3 runs to determine the confidence interval (CI).

(1) $$X_{\text{output}}=\frac{X-\min(X)}{\max(X)-\min(X)}$$

### 4.3 Third diagnosis layer

**Figure 7:**
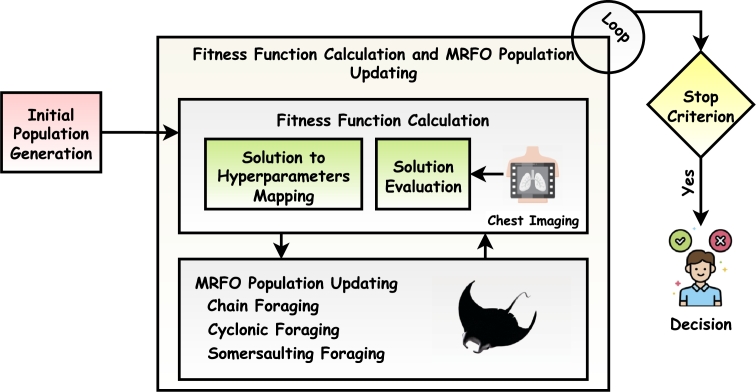
Graphical presentation of the internal phases on the third diagnosis layer.

In the third layer, the patient is projected to a chest X-ray (CXR). This scan is diagnosed after that and graded into four classes (i.e., “COVID-19”, “Normal”, “Lung Opacity”, “Viral Pneumonia”) which is considered a multiclass classification problem. This layer utilizes the transfer learning approach and metaheuristic optimization. The transfer learning is applied on five pre-train CNN model (i.e., VGG19, MobileNet, MobileNetV2, Xception, and DenseNet201). The utilized metaheuristic optimizer in the current study is the Manta-Ray Foraging Algorithm (MRFO) [64]. Normally, metaheuristic optimizers are used to find the best solution that minimizes (or maximizes) a problem. MRFO is used to determine the best hyperparameters combination that yields an excellent performance metric. Fig. 7 shows the internal phases on the third diagnosis layer.

**Figure 8:**
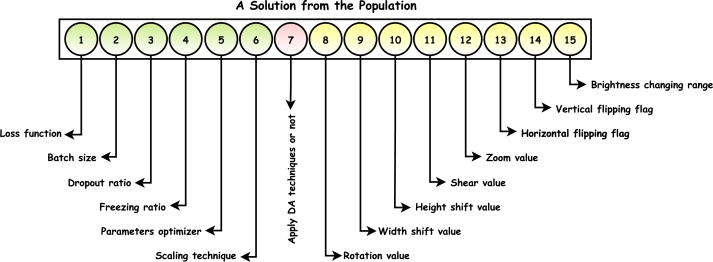
Graphical presentation of the contents of a solution from the population.

Initially, at the start of the optimization phase, the population is generated at random. The working population has a total of $N_{max}$ solutions. Each solution is represented as a vector of size (1×D), with each element in the range [0,1]. Every solution's cell represents a different learning hyperparameter where the solution indexing (from 1 to 15). Fig. 8 depicts a graphical representation of the hyperparameters. They can be partitioned into three parts (1) the major hyperparameters which are colored in light green, (2) the data augmentation (DA) flag which is colored in a very light shade of red and controls the third part, (3) the DA configurations which are colored in light yellow. If the DA flag is false, then the third part is ignored. We can derive from it that D=15 if data augmentation is used and D=7 otherwise.

After that, the fitness function score of each solution in the population is computed. It is divided into four steps: (1) conversion of hyperparameters, (2) model preparation, (3) training, and (4) model validation. The hyperparameters conversion process transforms the solution into the actual hyperparameters as shown in Fig. 8. The current study works in a restriced domain where each hyperparameter has a finite range of values. Table 3 shows the utilized ranges. After mapping each element in the solution to the associated hyperparameters, the target pre-trained TL model will be created using such hyperparameters. The pre-trained transfer learning models employed in this study are MobileNet, MobileNetV2, VGG19, Xception, and DenseNet201 with the “ImageNet” pre-trained weights. The dataset is divided into three subsets: training, testing, and validation. The training (and validation) to the testing ratio is 80% to 20%. In this study, the pre-trained TL model will start the training phase for a number of 7 epochs. The pre-trained transfer learning model is validated and generalized throughout the entire dataset. To judge the model's state-of-the-art performance, multiple performance indicators (e.g., accuracy, F1-score, precision, and recall) are reported.

**Table 3:** The range of each hyperparameter utilized in the current study.

Hyperparameter	Ranges
Loss function	Categorical Crossentropy
	Categorical Hinge
	KLDivergence
	Poisson
	Squared Hinge
	Hinge
Batch size	4 → 48 (step = 4)
Dropout ratio	[0 → 0.6]
Freezing ratio	1 → 100 (step = 1)
Parameters optimizer	Adam
	NAdam
	AdaGrad
	AdaDelta
	AdaMax
	RMSProp
	SGD
	Ftrl
	SGD Nesterov
	RMSProp Centered
	Adam AMSGrad
Scaling technique	L2-Normalization
	Standardization
	Min-Max
	Max-Absolute
Apply DA?	[Yes,No]
Rotation value	0^∘^ → 45^∘^ (step = 1^∘^)
Width shift value	[0 → 0.25]
Height shift value	[0 → 0.25]
Shear value	[0 → 0.25]
Zoom value	[0 → 0.25]
Horizontal flipping flag	[Yes,No]
Vertical flipping flag	[Yes,No]
Brightness changing range	[0.5 → 2.0]

The population solutions are sorted in descending order by fitness score after the performance metrics for each solution are computed, with the best solution at the top, to determine $X{\left(t\right)}_{best}$, which is then used in the rest of the procedure. In this stage, the population is updated using the MRFO equations. MRFO employs 3 strategies: chain foraging, cyclone foraging, and somersault foraging. MRFO believes that plankton, the primary food supply of manta rays, is the best item for chain foraging. Although the first individual seeks only food, the others desire both food and the people ahead of them in the chain. The MRFO behavior in cyclone foraging is similar to WOA [65], except that it not only spirals near to food but also follows the individuals in front of it. The exploration and exploitation phases of the MRFO switch based on the ratio of the current iteration to the maximum number of iterations $\frac{t}{T_{max}}$. When $\frac{t}{T_{max}}>rand$, the exploitation phase begins. If $\frac{t}{T_{max}}<rand$ is more than one, the approach switches to exploring. The somersault foraging occurs after either cyclone (or chain) foraging has been accomplished. Individual positions are updated by employing the greatest option available at the moment. In this phase, the food location serves as a pivot point. Individuals spin about the pivot, seeking a new spot. Equation (2) reflects the cyclone foraging process. The chain foraging process is reflects in Equation (3). The somersault foraging process is reflects in Equation (4) where $X_{i}(t)$ is the $i^{th}$ solution at iteration *t*, *α* (and *β*) are weight coefficients, *S* is the somersault factor, and *r* ($r_{1}$, and $r_{2}$) are random values ∈[0,1].

(2) $$X_{i}(t+1)=\left\{ \begin{array}{cc} X_{rand}+r\times \left(X_{rand}-X_{i}(t)\right)+\beta \times \left(X_{rand}-X_{i}(t)\right), & \text{if }((\frac{t}{T_{max}}<rand)\;\text{and}\;(i=1))\\ X_{rand}+r\times \left(X_{i-1}(t)-X_{i}(t)\right)+\beta \times \left(X_{rand}-X_{i}(t)\right), & \text{if }((\frac{t}{T_{max}}<rand)\;\text{and}\;(i>1))\\ X_{best}+r\times \left(X_{best}-X_{i}(t)\right)+\beta \times \left(X_{best}-X_{i}(t)\right), & \text{if }((\frac{t}{T_{max}}\geq rand)\;\text{and}\;(i=1))\\ X_{best}+r\times \left(X_{i-1}(t)-X_{i}(t)\right)+\beta \times \left(X_{best}-X_{i}(t)\right), & \text{if }((\frac{t}{T_{max}}\geq rand)\;\text{and}\;(i>1)) \end{array}\right.$$

(3) $$X_{i}(t+1)=\left\{ \begin{array}{cc} X_{i}(t)+r\times \left(X_{best}-X_{i}(t)\right)+\alpha \times \left(X_{best}-X_{i}(t)\right), & \text{if }(i=1)\\ X_{i}(t)+r\times \left(X_{i-1}(t)-X_{i}(t)\right)+\alpha \times \left(X_{best}-X_{i}(t)\right), & \text{Otherwise} \end{array}\right.$$

(4) $$X_{i}(t+1)=X_{i}(t)+S\times \left(r_{2}\times X_{best}-r_{3}\times X_{i}(t)\right)$$

**Figure 9:**
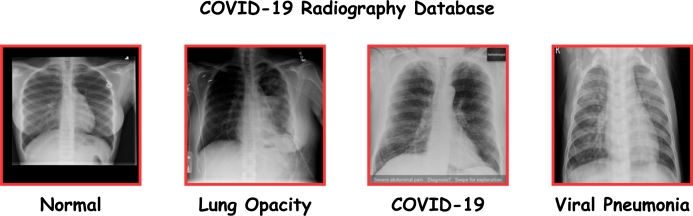
Sample from the “COVID-19 Radiography Database” [66]. From Left: Normal, Lung Opacity, COVID-19, and Viral Pneumonia cases.

To apply the suggested approach in the third diagnosis layer, the “COVID-19 Radiography Database” [66] dataset is used. A team of researchers from “Qatar University” in “Qatar”, and the “University of Dhaka” in “Bangladesh”, along with collaborators from “Pakistan” and “Malaysia” have produced it. It consists of fours classes: “COVID-19” (3,616 cases), “Normal” (10,192 cases), “Lung Opacity” (6,012 cases), “Viral Pneumonia” (1,345 cases). The reason behind focusing on this dataset is that it won the “COVID-19 Dataset Award” by “Kaggle Community”. The dataset is imbalanced but data augmentation is not applied before the splitting process to avoid data leakage. Sample from this dataset are shown in Fig. 9.

### 4.4 Performance evaluation

Multiple performance metrics are measured and reported from the confusion matrix (i.e., true positive, true negative, false positive, and false negative). They are (1) accuracy, (2) balanced accuracy, (3) precision, (4) sensitivity, (5) specificity, and (6) F1-score. Accuracy (Equation (5)) is the percentage of correct predictions made by the model out of all possible forecasts. Precision (Equation (6)) is commonly used by data scientists to overcome the constraints of accuracy. It indicates what percentage of positive forecasts are correct. Recall, like precision, (Equation (7)) seeks to determine the proportion of true positives that are accurately detected. Specificity, like recall (also known as sensitivity), (Equation (8)) seeks to determine what proportion of genuine negatives are properly detected. The F1-score (Equation (9)) represents the harmonic mean of precision and recall. When dealing with imbalanced data (i.e. when one of the target classes appears significantly more than the other), balanced accuracy can be used (Equation (10)) and it is the arithmetic mean of sensitivity and specificity.

(5) $$\text{Accuracy}=\frac{\text{TP}+\text{TN}}{\text{TP}+\text{TN}+\text{FP}+\text{FN}}$$

(6) $$\text{Precision}=\frac{\text{TP}}{\text{TP}+\text{FP}}$$

(7) $$\text{Sensitivity}=\frac{\text{TP}}{\text{TP}+\text{FN}}$$

(8) $$\text{Specificity}=\frac{\text{TN}}{\text{TN}+\text{FP}}$$

(9) $$\text{F1-Score}=\frac{2\times \text{Precision}\times \text{Sensitivity}}{\text{Recall}+\text{Precision}}$$

(10) Balanced Accuracy=0.5×(Sensitivity+Specificity)

### 4.5 Four diagnosis layer

**Figure 10:**
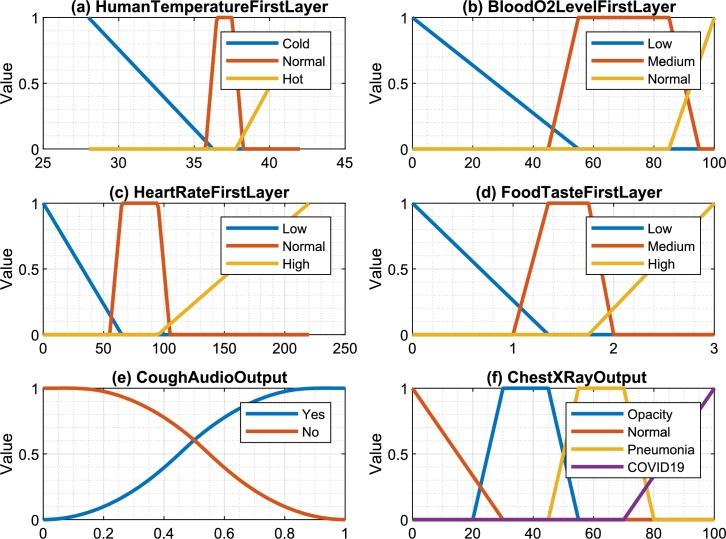
Graphical presentation of the input memberships for the fuzzy logic inference system in the four diagnosis layer. (a) Human Temperature (Input). (b) Blood O2 Level (Input). (c) Heart Rate (Input). (d) Food Taste (Input). (e) Cough Audio (Output). (f) Check X-Ray (Output).

In this layer, a fuzzy logic inference system is utilized based on the previous three layers to report a trusted (or accurate) decision. The Fuzzy Inference System is the central core of a fuzzy logic system, with decision making as its major function. It creates basic decision rules using the “IF-THEN” rules and connectors “OR” or “AND” [67]. The input memberships are presented in Fig. 10. The system reports three grades (1) low, (2) medium, and (3) high. A low output grade means that the patient has no COVID-19. A medium output grade means that there is a COVID-19 suspicion. A high output grade means that there is a COVID-19 disease. As noted in the first diagnosis layer, a patient can be initially assumed a COVID-19 patient if the temperature is high, the food taste is low (or medium), the blood O2 level is low (or medium), and the heart rate is high. From that, the first layer diagnosis rules can be summarized as in Table 4. It contains 27 critical rules while other rules are ignored or set to be “Low” output.

**Table 4:** Summarization of the rules in the fuzzy logic inference system in the four diagnosis layer.

#	Rule (If)	Rule (Then)
1	(XRay **is** COVID-19)	(Output **is** High)
2	(Cough **is** Yes) **and** (XRay **is** Pneumonia)	(Output **is** High)
3	(Temp **is** Hot) **and** (O2 **is** Low) **and** (HR **is** High) **and** (Taste **is** Low) **and** (Cough **is** No) **and** (XRay **is** Pneumonia)	(Output **is** High)
4	(Temp **is** Hot) **and** (O2 **is** Low) **and** (HR **is** High) **and** (Taste **is** Medium) **and** (Cough **is** No) **and** (XRay **is** Pneumonia)	(Output **is** High)
5	(Temp **is** Hot) **and** (O2 **is** Medium) **and** (HR **is** High) **and** (Taste **is** Medium) **and** (Cough **is** No) **and** (XRay **is** Pneumonia)	(Output **is** High)
6	(Temp **is** Hot) **and** (O2 **is** Medium) **and** (HR **is** High) **and** (Taste **is** Low) **and** (Cough **is** No) **and** (XRay **is** Pneumonia)	(Output **is** High)
7	(Temp **is** Hot) **and** (O2 **is** Low) **and** (HR **is** High) **and** (Taste **is** Low) **and** (Cough **is** No) **and** (XRay **is** Opacity)	(Output **is** Medium)
8	(Temp **is** Hot) **and** (O2 **is** Medium) **and** (HR **is** High) **and** (Taste **is** Low) **and** (Cough **is** No) **and** (XRay **is** Opacity)	(Output **is** Medium)
9	(Temp **is** Hot) **and** (O2 **is** Medium) **and** (HR **is** High) **and** (Taste **is** Medium) **and** (Cough **is** No) **and** (XRay **is** Opacity)	(Output **is** Medium)
10	(Temp **is** Hot) **and** (O2 **is** Low) **and** (HR **is** High) **and** (Taste **is** Medium) **and** (Cough **is** No) **and** (XRay **is** Opacity)	(Output **is** Medium)
11	(Temp **is** Normal) **and** (O2 **is** Low) **and** (HR **is** High) **and** (Taste **is** Low) **and** (Cough **is** No) **and** (XRay **is** Pneumonia)	(Output **is** Medium)
12	(Temp **is** Normal) **and** (O2 **is** Low) **and** (HR **is** High) **and** (Taste **is** Medium) **and** (Cough **is** No) **and** (XRay **is** Pneumonia)	(Output **is** Medium)
13	(Temp **is** Normal) **and** (O2 **is** Medium) **and** (HR **is** High) **and** (Taste **is** Medium) **and** (Cough **is** No) **and** (XRay **is** Pneumonia)	(Output **is** Medium)
14	(Temp **is** Normal) **and** (O2 **is** Medium) **and** (HR **is** High) **and** (Taste **is** Low) **and** (Cough **is** No) **and** (XRay **is** Pneumonia)	(Output **is** Medium)
15	(Cough **is** No) **and** (XRay **is** Normal)	(Output **is** Low)
16	(Cough **is** Yes) **and** (XRay **is** Opacity)	(Output **is** Medium)
17	(Cough **is** Yes) **and** (XRay **is** Normal)	(Output **is** Medium)
18	(Temp **is** Normal) **and** (O2 **is** Normal) **and** (HR **is** Normal) **and** (Taste **is** High) **and** (Cough **is** No) **and** (XRay **is** Normal)	(Output **is** Low)
19	(Temp **is** Cold) **and** (O2 **is** Normal) **and** (HR **is** Normal) **and** (Taste **is** High) **and** (Cough **is** No) **and** (XRay **is** Normal)	(Output **is** Low)
20	(Temp **is** Cold) **and** (O2 **is** Normal) **and** (HR **is** Normal) **and** (Taste **is** Medium) **and** (Cough **is** No) **and** (XRay **is** Normal)	(Output **is** Low)
21	(Temp **is** Cold) **and** (O2 **is** Medium) **and** (HR **is** Normal) **and** (Taste **is** High) **and** (Cough **is** No) **and** (XRay **is** Normal)	(Output **is** Low)
22	(Temp **is** Normal) **and** (O2 **is** Medium) **and** (HR **is** Normal) **and** (Taste **is** High) **and** (Cough **is** No) **and** (XRay **is** Normal)	(Output **is** Low)
23	(Temp **is** Normal) **and** (O2 **is** Normal) **and** (HR **is** High) **and** (Taste **is** High) **and** (Cough **is** No) **and** (XRay **is** Normal)	(Output **is** Low)
24	(Temp **is** Normal) **and** (O2 **is** Medium) **and** (HR **is** High) **and** (Taste **is** High) **and** (Cough **is** No) **and** (XRay **is** Normal)	(Output **is** Low)
25	(Temp **is** Normal) **and** (O2 **is** Low) **and** (HR **is** High) **and** (Taste **is** High) **and** (Cough **is** No) **and** (XRay **is** Normal)	(Output **is** Low)
26	(Temp **is** Hot) **and** (O2 **is** Normal) **and** (HR **is** Normal) **and** (Taste **is** High) **and** (Cough **is** No) **and** (XRay **is** Normal)	(Output **is** Low)
27	(Temp **is** Hot) **and** (O2 **is** Normal) **and** (HR **is** Normal) **and** (Taste **is** Medium) **and** (Cough **is** No) **and** (XRay **is** Normal)	(Output **is** Low)

## 5 Experiments and discussions

The experiments are performed in two sections. The first section discusses and reported the experiments related to the second layer while the second section handles the third layer. The working environment is Google Colab and the used programming language is Python.

### 5.1 Cough audio data experiments (second layer)

**Figure 11:**
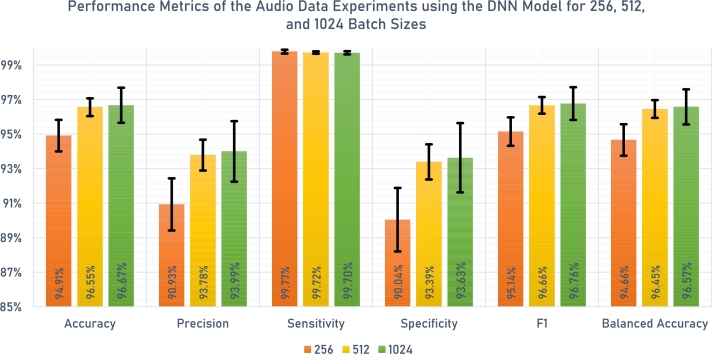
The performance metrics and their CIs concerning the audio dataset. The training hyperparameters are (1) 1,500 epochs, (2) 3 batch sizes, and (3) 5 runs to determine the CI.

As mentioned in the suggested approach in the second diagnosis layer, the “Cough Dataset” [53] is the used dataset. The training hyperparameters are (1) 1,500 epochs, (2) 3 batch sizes (i.e., 256, 512, and 1,024), and (3) 5 runs to determine the CI. Three batch sizes are tested to study the effect of increasing the batch size. The monitored performance metrics in this layer are (1) binary cross-entropy loss, (2) confusion metrics values, (3) accuracy, (4) precision, (5) sensitivity, (6) specificity, (7) F1-score, and (8) balanced accuracy. To get confidence result, the experiments are run for 5 times and the CI is measured for each metric. Table 5 shows the reported results concerning the audio dataset considering the five runs, the average values, and CI. From it, the mean accuracy is 94.91% while the suggested model is high sensitive to positive cases as it reports a mean sensitivity of 99.77% with a CI of ± 0.11%. The mean specificity to negative cases is 90.04% while the mean precision is 90.94%. The mean balanced accuracy is 94.66%. By increasing the batch size to 512, the reported results increased as reported in Table 6. By comparing the current results to the 256-batch size results, the mean accuracy, precision, specificity, F1, and balanced accuracy show increments by 1.65%, 2.86%, 3.34%, 1.52%, and 1.79% respectively. However, the mean sensitivity shows a low decrement by 0.05%. By increasing the batch size to 1,024, the reported results increased as reported in Table 7. By comparing the current results to the 256-batch size results, the mean accuracy, precision, specificity, F1, and balanced accuracy show increments by 1.76%, 3.07%, 3.59%, 1.62%, and 1.91% respectively. However, the mean sensitivity shows a low decrement by 0.07%. Also, by comparing the current results to the 512-batch size results, the increments are not very high and the CIs of the 512-batch size are smaller than the 1,024-batch size CIs. Hence a 512-batch size is considered a suitable choice. Fig. 11 shows the performance metrics and their CIs for the batch sizes 256, 512, and 1,024.

**Table 5:** The reported results concerning the audio dataset considering the five runs, the average values, and CI. The training hyperparameters are (1) 1,500 epochs, (2) batch size of 256, and (3) 5 runs to determine the CI.

Run #	Loss	TP	TN	FP	FN	Accuracy	Precision	Sensitivity	Specificity	F1	Balanced Accuracy
Run 1	0.1648	8,455	7,456	1,006	7	94.01%	89.37%	99.92%	88.11%	94.35%	94.01%
Run 2	0.1145	8,425	7,777	685	37	95.73%	92.48%	99.56%	91.90%	95.89%	95.73%
Run 3	0.1775	8,437	7,357	1,105	25	93.32%	88.42%	99.70%	86.94%	93.72%	93.32%
Run 4	0.1368	8,450	7,728	734	12	95.59%	92.01%	99.86%	91.33%	95.77%	95.59%
Run 5	0.1316	8,447	7,779	683	15	95.88%	92.52%	99.82%	91.93%	96.03%	95.88%
Mean	0.1450	8,442.8	7,619.4	842.6	19.2	94.91%	90.93%	99.77%	90.04%	95.14%	94.66%
CI	± 0.0201	± 9.3	± 155.6	± 155.6	± 9.3	± 0.91%	± 1.51%	± 0.11%	± 1.84%	± 0.82%	± 0.91%

**Table 6:** The reported results concerning the audio dataset considering the five runs, the average values, and CI. The training hyperparameters are (1) 1,500 epochs, (2) batch size of 512, and (3) 5 runs to determine the CI.

Run #	Loss	TP	TN	FP	FN	Accuracy	Precision	Sensitivity	Specificity	F1	Balanced Accuracy
Run 1	0.1002	8,435	7,898	564	27	96.51%	93.73%	99.68%	93.33%	96.62%	96.51%
Run 2	0.0816	8,432	8,030	432	30	97.27%	95.13%	99.65%	94.89%	97.33%	97.27%
Run 3	0.0872	8,451	7,963	499	11	96.99%	94.42%	99.87%	94.10%	97.07%	96.99%
Run 4	0.1146	8,436	7,735	727	26	95.55%	92.07%	99.69%	91.41%	95.73%	95.55%
Run 5	0.0998	8,438	7,886	576	24	96.45%	93.61%	99.72%	93.19%	96.57%	96.45%
Mean	0.0967	8,438.4	7,902.4	559.6	23.6	96.55%	93.78%	99.72%	93.39%	96.66%	96.45%
CI	± 0.0101	± 5.8	± 86.1	± 86.1	± 5.8	± 0.51%	± 0.89%	± 0.07%	± 1.02%	± 0.48%	± 0.51%

**Table 7:** The reported results concerning the audio dataset considering the five runs, the average values, and CI. The training hyperparameters are (1) 1,500 epochs, (2) batch size of 1,024, and (3) 5 runs to determine the CI.

Run #	Loss	TP	TN	FP	FN	Accuracy	Precision	Sensitivity	Specificity	F1	Balanced Accuracy
Run 1	0.0890	8,443	7,959	503	19	96.92%	94.38%	99.78%	94.06%	97.00%	96.92%
Run 2	0.0762	8,434	8,063	399	28	97.48%	95.48%	99.67%	95.28%	97.53%	97.48%
Run 3	0.0591	8,432	8,135	327	30	97.89%	96.27%	99.65%	96.14%	97.93%	97.89%
Run 4	0.1077	8,451	7,880	582	11	96.50%	93.56%	99.87%	93.12%	96.61%	96.50%
Run 5	0.1396	8,425	7,577	885	37	94.55%	90.49%	99.56%	89.54%	94.81%	94.55%
Mean	0.0943	8,437	7,922.8	539.2	25.0	96.67%	93.99%	99.70%	93.63%	96.76%	96.57%
CI	± 0.0242	± 7.9	± 169.7	± 169.7	± 7.9	± 1.02%	± 1.75%	± 0.09%	± 2.01%	± 0.95%	± 1.02%

**Figure 12:**
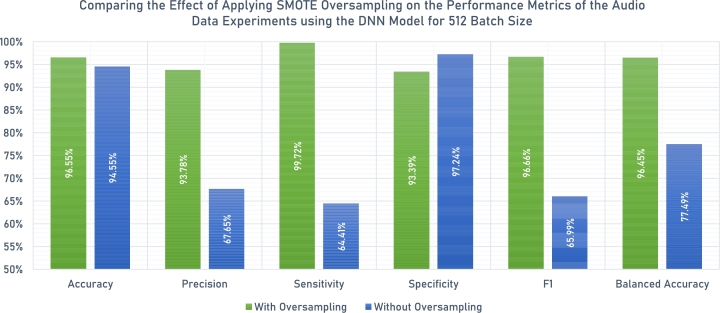
Comparing the effect of applying SMOTE oversampling on the performance metrics of the audio data experiments using the DNN model for 512 batch size.

To study the effect of oversampling using SMOTE using the cough dataset, the experiment is run again using a batch size of 512 but without oversampling. Table 8 shows the reported results without oversampling. It shows that without oversampling reports a decreased mean accuracy, precision, sensitivity, F1, and balanced accuracy by 2.01%, 26.13%, 35.31%, 30.67%, and 18.96% respectively. However, it reports an increased specificity by 3.86%. Hence, SMOTE oversampling is considered a suitable choice. Fig. 12 shows a graphical comparison between both of them.

**Table 8:** The reported results concerning the audio dataset (without oversampling) considering the five runs, the average values, and CI. The training hyperparameters are (1) 1,500 epochs, (2) batch size of 512, and (3) 5 runs to determine the CI.

Run #	Loss	TP	TN	FP	FN	Accuracy	Precision	Sensitivity	Specificity	F1	Balanced Accuracy
Run 1	1.4127	482	8,360	102	275	95.91%	82.53%	63.67%	98.79%	71.89%	81.23%
Run 2	0.9590	488	8,216	246	269	94.41%	66.49%	64.46%	97.09%	65.46%	80.78%
Run 3	0.9877	490	8,291	171	267	95.25%	74.13%	64.73%	97.98%	69.11%	81.35%
Run 4	0.8175	494	8,250	212	263	94.85%	69.97%	65.26%	97.49%	67.53%	81.38%
Run 5	0.9641	484	8,027	435	273	92.32%	52.67%	63.94%	94.86%	57.76%	79.40%
Mean	1.0282	487.6	8,228.8	233.2	269.4	94.55%	67.65%	64.41%	97.24%	65.99%	77.49%
CI	± 0.1765	± 3.7	± 97.9	± 97.9	± 3.7	± 1.07%	± 8.62%	± 0.49%	± 1.16%	± 4.19%	± 0.65%

### 5.2 X-ray scans data experiments (third layer)

**Figure 13:**
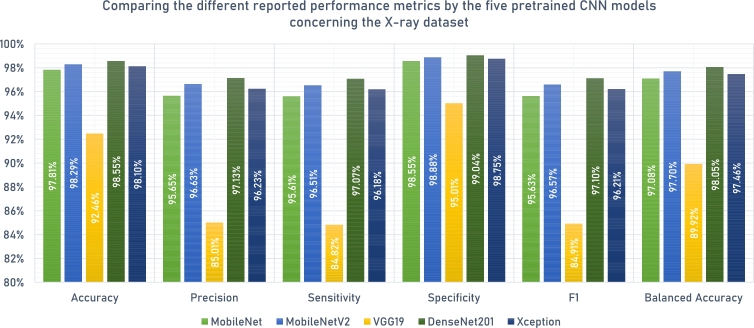
Comparing the different reported performance metrics by the five pretrained CNN models concerning the X-ray dataset.

As mentioned in the suggested approach in the third diagnosis layer, the “COVID-19 Radiography Database” [66] is the used dataset. The training hyperparameters are optimized using MRFO and their ranges are presented in Table 3. The used dataset is a multi-class problem and the output categories are “COVID-19”, “Normal”, “Lung Opacity”, “Viral Pneumonia”. The images are resized to (128×128×3). The number of MRFO solutions is set to 10 and the number of MRFO iterations is set also to 10. The number of epochs is set to 7 and the train to test split ratio is set to 80% to 20%. The pretrained CNN models use the “ImageNet” weights. The output activation function is set to SoftMax as the current problem is multi-class classification problem. The decision is made on the whole dataset accuracy. However, multiple performance metrics are captured and reported. Table 9 shows the reported results concerning the COVID-19 X-Ray dataset and five pretrained CNN models. It shows the optimized hyperparameters first and then the reported performance metrics second. It shows that (1) data augmentation is recommended by all models, (2) using a rotation angle below 20 is recommended by four models, (3) horizontal flipping is recommended by three models, (4) vertical flipping is not recommended by four models, (5) the DenseNet201 model reports the highest performance metrics, (6) the VGG19 model reports the lowest performance metrics, (7) the lowest loss value is 0.117 and is reported by the Xception model, (8) using a TF learning ratio above 50% is recommended by four models, and (9) using a dropout ratio above 40% is recommended by four models. Fig. 13 shows a graphical comparison between the different reported performance metrics by the five pretrained CNN models concerning the X-ray dataset.

**Table 9:** The reported results concerning the COVID-19 X-Ray dataset and five pretrained CNN models.

Model	MobileNet	MobileNetV2	VGG19	DenseNet201	Xception
TF Learn Ratio	30%	78%	98%	77%	57%
Loss	KLDivergence	Poisson	Squared Hinge	Categorical Crossentropy	Categorical Crossentropy
Batch Size	48	36	24	20	24
Dropout	0.15	0.37	0.6	0.12	0.28
Optimizer	NAdam	SGD Nesterov	AdaDelta	SGD Nesterov	Adam
Scaling Technique	Max. Normalization	Max. Normalization	N/A	Robust	Min-Max
Apply Augmentation	Yes	Yes	Yes	Yes	Yes
Rotation Range	33	18	14	1	16
Width Shift Range	0.15	0.16	0.16	0.18	0.17
Height Shift Range	0.21	0.23	0.2	0.03	0.01
Shear Range	0.24	0.23	0.1	0.18	0.22
Zoom Range	0.04	0.24	0.22	0.23	0.02
Horizontal Flip	No	No	Yes	Yes	Yes
Vertical Flip	No	No	Yes	No	No
Brightness Range	0.9-1.91	0.78-1.21	0.74-1.32	1.09-1.46	1.25-1.93
Loss	0.148	0.279	0.896	0.144	0.117
TP	20,192	20,395	17,934	20,539	20,337
TN	62,441	62,685	60,269	62,873	62,636
FP	919	711	3,163	607	796
FN	928	737	3,210	621	807
Accuracy	97.81%	98.29%	92.46%	98.55%	98.10%
Precision	95.65%	96.63%	85.01%	97.13%	96.23%
Sensitivity	95.61%	96.51%	84.82%	97.07%	96.18%
Specificity	98.55%	98.88%	95.01%	99.04%	98.75%
F1	95.63%	96.57%	84.91%	97.10%	96.21%
Balanced Accuracy	97.08%	97.70%	89.92%	98.05%	97.46%

The experimental results demonstrate that the proposed framework is trustworthy and effective in terms of accuracy, precision, sensitivity, specificity, F1-score, and balanced accuracy as follows: (1) audio-based classification: 96.55%, 93.78%, 99.72%, 93.39%, 96.66%, and 96.45% respectively. (2) CXR-based classification: The DenseNet201 reports 98.55%, 97.13%, 97.07%, 99.04%, 97.10%, and 98.05% respectively. The accuracy achieved in this study is comparable to the accuracies reported in other studies that used CXR images, such as Ahmadian et al. [9], Ardakani et al. [33], Khan et al. [34], Rajawat et al. [35], Swarup and Anupam [10], and Ranjan et al. [39]. The study also used a CNN-based model, which is a commonly used model for image classification tasks. Overall, the study's results are consistent with the current literature in the field of COVID-19 classification using deep learning techniques. The study's high accuracy suggests that deep learning models can be a useful tool for COVID-19 diagnosis and screening. However, it is important to note that these models should be used in conjunction with other diagnostic methods to improve the accuracy of COVID-19 diagnosis.

### 5.3 Clinical and technical outlooks

The clinical outlook for the proposed algorithm is positive as it introduces a four-layer diagnostic framework for COVID-19 that is flexible, automated, and non-invasive. The method utilizes multiple modalities, such as temperature, blood oxygen levels, breathing patterns, cough sounds, and chest X-rays, achieving high accuracy in COVID-19 diagnosis. Furthermore, the system has the potential to minimize the risk of virus transmission as it does not require close contact between patients and healthcare providers, which is crucial during the ongoing COVID-19 pandemic.

In terms of the technical outlook, the proposed four-layer diagnostic framework for COVID-19 is based on machine learning techniques such as CNN, TL, FIS, and MRFO algorithms. The CNN architecture extracts features from input data, and TL fine-tunes the CNN model for optimal performance. The FIS algorithm handles uncertainty and imprecision, and MRFO integrates the outputs of different diagnosis layers. Performance metrics show high accuracy, sensitivity, and specificity in diagnosing COVID-19, and the CNN model's hyperparameters are automatically determined, making the method flexible. Overall, the technical outlook of the system is promising, and it has the potential to accurately and efficiently diagnose COVID-19 using multimedia vital signs.

## 6 Limitations

The suggested non-invasive diagnostic framework has some limitations that should be considered. Firstly, the framework employs two datasets that may not be representative of the global population, which could limit the accuracy and applicability of the model. Secondly, the research outcomes were derived from a controlled environment and have not been tested in real-world medical scenarios, so the model's performance may differ in clinical settings. Thirdly, the proposed framework relies on multiple modalities, including temperature, blood oxygen levels, breathing patterns, cough sounds, and chest X-rays, which could increase the complexity of the diagnostic process and require additional resources. It is important to note that obtaining consistent data from the same patient for all four phases was not feasible in the current research, and as a result, the fourth phase was not evaluated. However, a detailed description of the fourth phase is provided in Subsection 4.5 of the manuscript.

## 7 Conclusions

The COVID-19 pandemic impacts society and dramatically affects healthcare centers. Deep learning advancements have significantly aided in proper COVID-19 control and management. Cough sounds and medical image modalities of real-life patient datasets are used for training deep-learning models to diagnose COVID-19. However, relying on a single modality cannot accurately detect the virus. This study suggests a multimodal classification framework that consists of four layers. The first layer is the diagnosis layer that captures the elementary diagnosis of patients, such as Heart Rate, Blood Oxygen Level, Temperature, Food Taste, and Breathing Profile. The second layer is Diagnosis Layer which applies audio features extraction on the coughing profile, preprocesses the features, and classifies them using a deep neural network. The patient is projected to a chest X-ray in the third layer. Following that, the scan is diagnosed and classified into four classes (i.e., “COVID-19”, “Normal,” “Lung Opacity,” and “Viral Pneumonia”). A fuzzy logic inference system in the fourth layer is used to report a trusted (or accurate) decision based on the previous three layers. Two datasets were used to apply the suggested approach (1) “Cough Dataset contains 3,805 train and 5,732 test records. (2) “COVID-19 Radiography Database “dataset consists of 21,165 images in four classes. The experimental results demonstrate that the proposed framework is trustworthy and effective in terms of accuracy, precision, sensitivity, specificity, F1-score, and balanced accuracy as follows: (1) audio-based classification: 96.55%, 93.78%, 99.72%, 93.39%, 96.66%, and 96.45% respectively. (2) CXR-based classification: The DenseNet201 reports 98.55%, 97.13%, 97.07%, 99.04%, 97.10%, and 98.05% respectively.

## CRediT authorship contribution statement

Saleh Ateeq Almutairi contributed to all aspects of this research and manuscript. Specifically, he: (1) conceived and designed the study, (2) conducted the experiments and collected the data, (3) analyzed and interpreted the data, (4) drafted and revised the manuscript, and (5) approved the final version of the manuscript for submission.

## Declaration of Competing Interest

The author, Saleh Ateeq Almutairi, certifies that he has NO conflict of interest with another person or organization that might influence the work of this study.

## Data Availability

The proposed approach in the second diagnostic layer utilizes the “Cough Dataset” [53] as the chosen dataset. Additionally, the suggested approach in the third diagnostic layer involves using the “COVID-19 Radiography Database” [66] as the selected dataset.
